# Pain Management with Natural Products: Neurophysiological Insights

**DOI:** 10.3390/ijms26136305

**Published:** 2025-06-30

**Authors:** Mamoru Takeda, Yukito Sashide

**Affiliations:** Laboratory of Food and Physiological Sciences, Department of Life and Food Sciences, School of Life and Environmental Sciences, Azabu University, 1-17-71, Fuchinobe, Chuo-ku, Sagamihara 252-5201, Kanagawa, Japan; sashide012@hotmail.co.jp

**Keywords:** natural compounds, complementary alternative medicine, nociceptive pain pathological pain, neurophysiology, local anesthesia, intravenous anesthesia, non-steroidal anti-inflammatory drugs, inflammation

## Abstract

Recently, complementary and alternative medicine have been actively employed for patients experiencing symptoms unresponsive to Western medical treatments like drug therapy. Natural compounds, including polyphenols, carotenoids, and omega fatty acids, have demonstrated various beneficial biological actions for human health in several studies. Given their broad pharmacological activities and reduced toxicity, these compounds possess significant potential as resources for the development of natural analgesic drugs. Given recent studies showing that natural compounds can modulate neuronal excitability (including nociceptive sensory transmission through mechanoreceptors and voltage-gated ion channels) and inhibit the cyclooxygenase-2 cascade, these compounds hold promise as complementary and alternative medicine candidates, particularly as therapeutic agents for nociceptive and pathological pain. This review focuses on elucidating the mechanisms by which natural compounds modulate neuronal electrical signals—including generator potentials, action potentials, and postsynaptic potentials—in nociceptive pathway neurons, potentially leading to local and intravenous anesthetic effects, as well as inflammatory pain relief. Specifically, we discuss the contribution of natural compounds to the relief of nociceptive and/or pathological pain and their potential clinical application, drawing on our recent published in vivo studies.

## 1. Introduction

Given the limitations of Western medical treatments, including drug therapy, for some patients with specific symptoms, complementary and alternative medicine (CAM) has been increasingly employed and holds promise for the treatment of chronic pain [1,2,3]. Current Western medicine defines CAM as a medical system lacking rigorous scientific testing and widespread clinical application. Commonly, CAM is primarily associated with herbal medicines and acupuncture. Evidence from several studies indicates that natural compounds possess various beneficial biological actions for human health, including, but not limited to, anti-oxidative, anti-inflammatory, and cardioprotective effects, and also antinociception [4,5,6,7,8].

Recognizing the multitude of adverse effects resulting from the long-term administration of analgesic drugs, addressing the urgent need to substitute them with natural compounds that lack toxic side effects while offering significant curative benefits is crucial in clinical treatment. The significant potential of natural compounds as resources for the development of natural analgesic drugs stems from their broad pharmacological activities and reduced toxicity. It is well established that currently used local anesthetics and anti-inflammatory analgesics in medicine for pain relief are associated with side effects alongside their intended actions. Furthermore, inflammation is known to reduce the efficacy of local anesthetics, particularly dental anesthetics [9]. Despite the potent and effective cyclooxygenase-2 (Cox-2) inhibitory action of non-steroidal anti-inflammatory drugs (NSAIDs) for pain relief, the pharmacological treatment of pain with NSAIDs is unfortunately associated with several adverse effects, such as stomach ulcers and myocardial infarction [10]. Consequently, natural compounds, including phytochemicals and fatty acids, are anticipated to be promising candidates within CAM, offering the potential for pain relief in inflamed tissues without the side effects commonly linked to traditional pain medications. Various natural products derived from vegetables/fruits and marine animals include polyphenols (resveratrol, chlorogenic acid, isoflavone, catechin, naringenin, quercetin, and myricetin), carotenoids (lutein, astaxanthin), amino acids (theanine), omega fatty acids (docosahexaenoic acid), and fatty acids (decanoic acid). Examples of the natural sources of these compounds include resveratrol in grape seeds and skins; chlorogenic acid in coffee beans; isoflavones in soybeans; quercetin in onions; and catechin in green tea. Lutein, a naturally occurring carotenoid, is present in fruits and leafy vegetables. Theanine, an amino acid derived from glutamic acid, is found almost exclusively in certain teas. Consequently, these organic compounds are commonly found in foods like fruits and vegetables that are part of our regular diet.

Although previous review articles have summarized animal studies suggesting the potential of several natural compounds, such as phytochemicals and fatty acids, to relieve pathological pain (encompassing inflammatory and neuropathic pain) [11,12,13], there is a scarcity of reviews detailing the neurophysiological mechanisms through which natural compounds modulate the excitability of nociceptive neurons within the pain pathway during nociceptive and pathological states, employing in vivo electrophysiological methods. Recent in vitro studies demonstrate that natural compounds, including phytochemicals and fatty acids, can modulate neuronal excitability in the nervous system. Their mechanisms involve effects on nociceptive sensory transmission (via mechanoreceptors and voltage-gated ion channels), neurotransmitter receptors, and the inhibition of the cyclooxygenase-2 (Cox-2) cascade across various tissues. These findings suggest natural compounds hold promise as complementary and alternative medicine (CAM) candidates, especially as therapeutic agents for nociceptive and pathological pain [14,15,16,17,18,19,20,21,22,23,24,25,26,27,28,29,30,31,32,33,34,35,36,37,38,39,40,41,42,43,44,45,46,47,48,49,50,51,52]. Table 1 presents a compilation of the modulatory effects of natural compounds on their potential molecular targets in diverse tissues, including neuronal cells, based on in vitro experimental findings. Following these in vitro experimental findings, we recently conducted animal studies in our laboratory using a neurophysiological approach to investigate the pain-relieving effects of natural compounds present in foods. Our published research has shown the following effects of natural compounds: (i) local anesthetic effect on nociceptive pain; (ii) intravenous anesthetic effect on nociceptive pain; (iii) local anesthetic effect on acute inflammatory pain; and (iv) anti-inflammatory effect on chronic pain relief. In this review, we aim to discuss the contribution of natural compounds to the relief of nociceptive and/or pathological pain and their potential clinical application, informed by our recent published in vivo studies [53,54,55,56,57,58,59,60,61,62,63,64,65,66,67,68,69,70,71,72,73,74,75,76].

## 2. Clinical Manifestations of Nociceptive and Pathological Pain

Congenital analgesia, a genetic disease involving deficient nociceptors [77], results in a complete inability to feel pain. Consequently, affected individuals often suffer from joint deformities due to repeated fractures and face a persistent threat of life-threatening disabilities, including tissue necrosis and sepsis stemming from burns. This emphasizes that pain functions as a vital “biological warning signal”, safeguarding the body against tissue damage resulting from nociceptive stimuli and highlighting its essential role as a sensation for our survival [78]. Physiological pain, also known as nociceptive pain, generally acts as a warning signal. However, pathological pain represents a state where this “biological warning signal” function is lost. It is characterized by changes in the neurons of the pain transmission pathway and sustained activation of signal transduction, leading to chronic pain that severely diminishes quality of life, often persisting even after significant tissue damage has healed [78]. Pathological pain, which no longer acts as a warning signal, includes conditions like inflammatory pain and neuropathic pain. Inflammatory pain specifically results from the sensitization of nociceptors by inflammatory chemicals (for example, prostaglandin E2 [PGE2]) at areas of tissue damage, such as burns and joint pain [79]. Neuropathic pain is a consequence of nerve damage and is characterized by its persistence even after the initial injury has resolved, a phenomenon observed in clinical dentistry following procedures like tooth extractions and implants. Key examples of neuropathic pain include diabetic neuropathy, sciatica, and trigeminal neuralgia. Hyperalgesia and allodynia are frequently reported symptoms associated with these pathological pain states [80,81]. Hyperalgesia involves an increased sensitivity to pain stimuli, and allodynia is pain evoked by stimuli that are not normally painful [82]. It is hypothesized that these pathological pain conditions result from plastic changes in the nature of sensory neurons within somatic sensory pathways, often stemming from inflammation or injury to peripheral tissues [79]. Peripheral sensitization, which typically includes heightened excitability of peripheral nerve endings and chemical substance-mediated communication between neurons and between neurons and glial cells in sensory ganglia resulting from tissue inflammation or injury, is generally understood to initiate central sensitization, including hyperalgesia [83,84,85,86].

## 3. A Comprehensive Overview of the Trigeminal Pain Transmission Pathway

**Figure 1:**
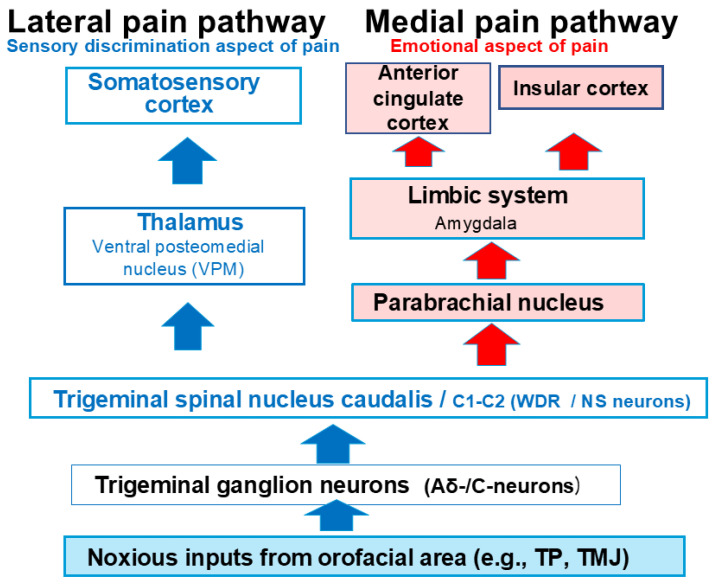
The trigeminal nociceptive sensory pathway comprises lateral and medial systems. While the lateral system transmits information related to the sensory-discriminative aspects of pain, the medial system conveys information about the emotional aspects. Orofacial noxious stimuli are relayed from Aδ- and C-trigeminal ganglion neurons to the spinal trigeminal nucleus caudalis (SpVc) and the upper cervical dorsal horn (C1–C2). Neurons in the SpVc and C1–C2 project axons to the somatosensory cortex via the ventral posteromedial nucleus of the thalamus (lateral pathway) and also to the anterior cingulate cortex and the insular cortex via limbic structures such as the amygdala (medial pathway). TP = tooth pulp, TMJ = temporomandibular joint.

This review focuses on the potential contribution of natural compounds to the relief of nociceptive and pathological pain. Drawing upon our recent findings that demonstrate the effects of natural compounds on the excitability of trigeminal nociceptive neurons in a rat model of trigeminal pain, we will first introduce the trigeminal pain pathway and the general properties of nociceptive neurons. Figure 1 provides a schematic representation of the trigeminal nociceptive sensory pathway, detailing both the lateral and medial divisions. The lateral pain system is responsible for relaying sensory input concerning the discriminative features of pain, notably, its location and intensity. In contrast, the medial pain system mediates the affective and emotional components of the pain experience. The trigeminal nervous system is characterized by distinct anatomical organizations and physiological mechanisms dedicated to the processing of orofacial nociceptive inputs alongside non-noxious sensory information. Specifically, the oral mucosal membrane, tongue, tooth pulp, periodontal tissue, and temporomandibular joint (TMJ) receive innervation from small-diameter myelinated Aδ-fibers and unmyelinated C-fibers, which are instrumental in the transmission and perception of orofacial nociception [83]. Nociceptive sensory input originating from the region innervated by trigeminal ganglion (TG) neurons is transmitted from trigeminal afferent fibers to second-order neurons located in the spinal trigeminal nucleus (SpV) of the brainstem and the upper cervical spinal cord (C1–C2) [81,82]. The SpV, a pivotal relay center in the processing and transmission of orofacial sensory information, exhibits a functional organization comprising three distinct nuclei: oralis, interpolaris, and caudalis [81,82]. Analogous to the dorsal horn of the C1–C2, the SpV caudalis (SpVc) acts as a key relay site for trigeminal nociceptive signals originating from inflammation and tissue damage [81,82]. Subsequently, projection neurons located within the SpVc and the C1–C2 segments of the spinal cord extend axonal projections to the thalamic nuclei, specifically, the ventroposteromedial (VPM) and medial thalamic nuclei, as well as to the parabrachial nucleus (PBN) [87,88,89,90]. Nociceptive neurons that receive noxious sensory inputs from the orofacial region are somatotopically organized within the VPM thalamic nucleus; however, this precise spatial arrangement is not observed in the medial thalamic nuclei or the parabrachial nucleus (PBN). Specifically, noxious stimuli originating from intraoral structure are relayed to the somatosensory cortex through the medial division of the VPM, whereas nociceptive signals from the face and head are conveyed via the lateral division of the VPM [90]. Nociceptive neurons within the VPM thalamic nucleus that are innervated by afferents from the orofacial region extend axonal projections to the primary (SI) and secondary (SII) somatosensory cortical areas. Conversely, afferent signals directed towards the medial thalamic nuclei and the PBN project to limbic cortical regions, notably, the anterior cingulate cortex (ACC) and the insular cortex (INS) [91]. Neurons within the INS receive both sympathetic and parasympathetic autonomic afferents and play a role in the modulation of autonomic functions. Nevertheless, the precise response characteristics of nociceptive neurons within the INS are not yet fully elucidated. Despite the established involvement of these cortical regions in pain perception, the underlying mechanisms governing the contribution of these higher brain areas to the overall pain experience remain largely obscure.

Chronic pathological states, including tissue inflammation, are capable of inducing alterations in the characteristics of somatic sensory pathways, consequently leading to the development of hyperalgesia and allodynia [81,82]. Furthermore, the processing of sensory information within the spinal trigeminal nucleus or at supraspinal levels is modulated by changes in the excitability of primary afferent neurons, a process referred to as peripheral sensitization [81,82]. The SpVc and the C1–C2 segments of the spinal cord contain two distinct populations of nociceptive neurons, which are differentiated based on their responsiveness to mechanical stimuli applied to the orofacial area. These populations comprise nociceptive-specific (NS) neurons, predominantly situated in the superficial laminae I–II, and wide-dynamic-range (WDR) neurons, primarily found within the deeper laminae IV–V [81,82]. Nociceptive-specific (NS) neurons exhibit a selective response solely to noxious stimuli, such as high-threshold mechanical stimulation, applied within their receptive fields. This suggests that NS neurons relay signals encoding the precise location of the stimulus to supraspinal brain regions [84]. Conversely, WDR neurons demonstrate responsiveness to both noxious stimuli (mediated by Aδ- and C-fibers) and non-noxious stimuli (mediated by Aβ-fibers), and are characterized by extensive receptive fields [81,82]. Graded application of nociceptive stimuli to the region of maximal sensitivity within the receptive field resulted in a stimulus intensity-dependent increase in the firing rate of WDR neurons in the SpVc. Furthermore, a substantial number of these WDR neurons also exhibited responsiveness to noxious thermal stimulation, implying that these neurons relay intensity-encoded information to supraspinal brain regions. Given that SpVc and C1–C2 WDR neurons can receive convergent inputs from tooth pulp, facial skin, jaw, masseter muscle, and phrenic afferents [81], it is plausible that WDR neurons contribute to the phenomenon of referred pain, where pain is perceived at a location distinct from the origin of the painful stimulus. Previous studies have demonstrated (i) that WDR neurons in the SpVc responding to whisker pad stimulation play a crucial role in the mechanisms underlying mechanical hyperalgesia associated with orofacial inflammatory pain [70,71,72,73,74,75], and (ii) the possibility of NS neurons transforming into WDR neurons following Complete Freund’s Adjuvant (CFA) inflammation [84,85]. Therefore, our study focused on the effects of natural compounds primarily on the nociceptive activity of SpVc WDR neurons rather than NS neurons.

## 4. The Role of Electrical and Chemical Signals in Pain Transmission: From Periphery to Brain

**Figure 2:**
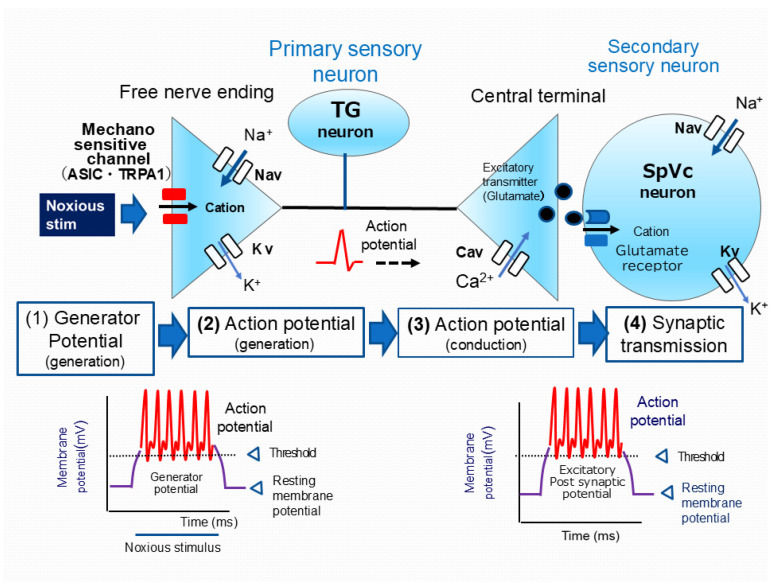
Trigeminal nociceptive sensory transduction involves a precise signaling cascade. When noxious mechanical stimulation is applied to the tissue, it activates a generator potential in the peripheral terminal of trigeminal ganglion (TG) neurons. This depolarization subsequently opens Nav and Kv channels, thereby generating action potentials. These action potentials are conducted through primary afferent TG neurons to the central terminal in nociceptive neurons within the spinal trigeminal subnucleus caudalis (SpVc). Here, presynaptic Cav channels open, leading to the release of neurotransmitters into the synaptic cleft. These neurotransmitters then bind to postsynaptic ionotropic glutamate receptors, activating excitatory postsynaptic potentials (EPSPs). If the EPSP amplitude surpasses the action potential threshold, a barrage of action potentials is conducted to higher centers in the pain pathway, resulting in pain perception. TG = trigeminal ganglion, Nav = voltage-gated sodium channel, Kv = voltage-gated potassium channel, Cav = voltage-gated calcium channel, SpVc = spinal trigeminal nucleus caudalis, EPSP = excitatory postsynaptic potential, ASIC = acid-sensing ion channel, TRPA1 = transient receptor potential ankyrin 1.

Primary afferent nerve fibers, including TG neurons, encompass myelinated Aδ-fibers (thin, slow-conducting) and unmyelinated C-fibers, both crucial for pain transmission [81,82]. Aδ-fibers mediate well-localized, sharp, “fast” pain, whereas C-fibers transmit poorly localized, dull, “slow” pain [81,82]. TG neurons, characterized by their pseudo-bipolar morphology, possess a central axon that establishes chemical synaptic connections with secondary neurons. In contrast, their peripheral axon forms a nociceptive free nerve terminal [81,82]. Nociceptors function as energy transducers, converting external noxious stimuli—thermal, cold, mechanical, or chemical energy—into electrical signals [80]. As shown in Figure 2, the processing of sensory information in primary afferent fibers can be broadly categorized into four stages: (i) transduction—peripheral terminals convert external stimuli; (ii) action potential generation and initiation; (iii) action potential propagation along conducting neurons; and (iv) transmission—central terminals form the presynaptic component of the initial synapse in the sensory pathways of the central nervous system (CNS) [81,92].

Upon application of a nociceptive mechanical stimulus to the peripheral receptive field of the skin, transient receptor potential ankyrin 1 (TRPA1) and acid-sensing ion channels (ASICs), which are candidate nociceptive mechanosensitive ion channels expressed on free nerve endings, are activated. This activation results in an increased permeability of the cell membrane to cations, leading to an inward current and the generation of a depolarizing generator potential [93,94,95,96]. Serving as an analog signal with an amplitude proportional to stimulus intensity, the non-propagating generator potential can initiate a digital action potential, governed by the all-or-none principle. Consequently, generator potentials in nociceptors of primary sensory neurons are known as “triggering potentials” because they initiate the action potential. When the membrane potential at free nerve terminals, in response to noxious stimuli, reaches the threshold for action potential firing, it triggers an action potential. This rapid electrical event consists of a depolarization phase, mediated by the opening of voltage-gated sodium (Nav) channels and the resulting influx of Na^+^ ions, followed by a repolarization phase, driven by the opening of voltage-gated potassium (Kv) channels and the subsequent efflux of K^+^ ions [81,92]. Nociceptive neurons express both tetrodotoxin-sensitive (TTX-S) and tetrodotoxin-resistant (TTX-R) Nav channels. Aδ neurons express both TTX-S and TTX-R Nav channels, whereas C-neurons primarily express TTX-R Nav channels [97].

Noxious stimuli applied to the receptive field elicit generator potentials whose amplitude is proportional to the stimulus intensity, subsequently modulating the firing frequency of action potentials [81,82]. Action potentials, initiated at peripheral free nerve endings, are actively propagated along the axon to the central terminal by the sequential opening and closing Nav and Kv channels. Depolarization of the central nerve terminal upon action potential arrival activates voltage-gated calcium (Cav) channels, resulting in an influx of calcium ions (Ca^2+^). Elevated intracellular Ca^2+^ levels at the presynaptic terminal induce the release of excitatory neurotransmitters, including glutamate, into the synaptic cleft. These neurotransmitters then bind to ionotropic glutamate receptors on secondary sensory neurons, causing cation influx and subsequent neuronal excitation. Cation influx resulting from the activation of glutamate receptors generates an excitatory postsynaptic potential (EPSP). Depolarization caused by the EPSP can initiate an action potential in the postsynaptic neuron if it reaches the firing threshold. The amount of neurotransmitter released is thought to determine the amplitude of the EPSP. Subsequently, the frequency of action potential firing, which correlates with the EPSP amplitude, is processed by higher CNS centers as encoding pain intensity [81,82].

## 5. Modulation of Nociceptive Transmission by Natural Compounds

### 5.1. Natural Compounds as Local Anesthetics: Targeting Nociceptive Pathways

Yu et al. [15] previously demonstrated that the phytochemical resveratrol dose-dependently suppresses TRPA1-mediated currents, suggesting its potential to inhibit generator potentials in sensory terminals. Furthermore, prior research indicates that resveratrol dose-dependently modulates Nav and Kv channel currents in primary sensory neurons of the spinal dorsal root ganglia (DRG) [16,17]. These results suggest that resveratrol could attenuate the generator potential and consequently reduce the action potential firing rate in primary sensory neurons activated by noxious stimuli, potentially mimicking the effects of the local anesthetic lidocaine. However, the clinical utility of lidocaine is limited by its known cardiovascular, central nervous system, and cardiac side effects, as well as its diminished effectiveness in inflammatory conditions [9]. In anesthetized rats, we recently showed that local subcutaneous application of resveratrol to the receptive field dose-dependently and reversibly suppressed the responsiveness of SpVc WDR neurons to both non-noxious and noxious mechanical stimulation [53]. Notably, the magnitude of this inhibitory effect was nearly identical to that observed with a clinically relevant concentration of 1% lidocaine. These findings suggest that local resveratrol administration to the peripheral receptive field inhibits TRPA1 and Nav channels while facilitating Kv channel opening in the peripheral free nerve endings of TG neurons, thereby reducing the excitability of SpVc WDR neurons. This strongly implies that resveratrol could serve as a substitute for local anesthetics, potentially without the associated cardiovascular side effects [98].

Previous studies repored that the phytochemical chlorogenic acid facilitates Kv channel opening and inhibits ASIC channel currents in acutely isolated TG neurons under in vitro conditions [21,22]. Following the in vitro findings, we examined the in vivo effects of topical chlorogenic acid on the responsiveness of SpVc WDR neurons in anesthetized rats to both non-noxious and nociceptive mechanical stimuli. Our results demonstrated a dose-dependent and reversible inhibition of neuronal excitability by chlorogenic acid, which appeared to be mediated by the suppression of both generator and action potential initiation, indicative of a local anesthetic-like effect [54]. Notably, in vitro studies on acutely isolated TG neurons have revealed that the isoflavone genistein elicits a significant and concentration-dependent reversible suppression of both TTX-R and TTX-S Nav channel currents [23]. Thus, our recent in vivo study demonstrated that local genistein delivery to the receptive field concentration-dependently reduced the excitability of rat SpVc WDR neurons. Notably, a 10 mM concentration of genistein exhibited an inhibitory efficacy similar to that of 1% (37 mM) lidocaine [55], and our findings also suggest the potential for genistein to partially replace lidocaine in clinical applications, potentially reducing the required dose of the conventional anesthetic by half. In vitro whole-cell voltage clamp studies by Kim et al. [29] demonstrated that (-)-epigallocatechin-3-gallate (EGCG) dose-dependently inhibits currents through both TTX-R and TTX-S Nav channels in DRG neurons. Consistent with in vitro findings, our in vivo extracellular electrophysiological recordings revealed that local application of (-)-epigallocatechin-3-gallate suppressed the excitability of nociceptive SpVc neurons, likely through modulation of Nav and Kv channels in the TG [56]. These results suggest that (-)-epigallocatechin-3-gallate may represent a novel local anesthetic for trigeminal nociceptive pain management with a potentially improved safety profile.

**Figure 3:**
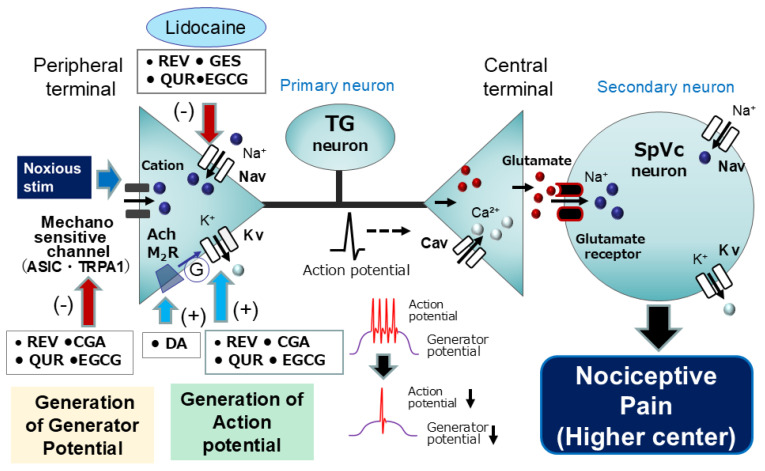
Potential molecular targets for natural compounds as local anesthetic agents include mechanosensitive ionic channels (ASIC and TRPA1), Nav channels, and Kv channels. When locally applied to peripheral tissues, natural compounds (resveratrol, chlorogenic acid, genistein, quercetin, (-)-epigallocatechin-3-gallate, and decanoic acid) inhibit the generation of generator potentials and/or action potentials in the peripheral terminals of primary afferents following nociceptive stimulation by blocking ASICs and TRPA1, inhibiting Nav channels, and facilitating Kv channels. TG = trigeminal ganglion, Nav = voltage-gated sodium channel, Kv = voltage-gated potassium channel, Cav = voltage-gated calcium channel, SpVc = spinal trigeminal nucleus caudalis, (+) = facilitation, (−) = inhibition, ASIC = acid-sensing ion channel, TRPA1 = transient receptor potential ankyrin 1, Lidocaine = local anesthetic agent, REV = resveratrol, CGA = chlorogenic acid, GES = genistein, QUR = quercetin, EGCG = (-)-epigallocatechin-3-gallate, DA = decanoic acid, G = G-protein.

However, prior investigations into the local anesthetic effects of natural compounds, namely, resveratrol, chlorogenic acid, genistein, and (-)-epigallocatechin-3-gallate, on nociception have primarily assessed alterations in the excitability of nociceptive secondary neurons, without direct examination of primary afferent activity. Consequently, the question of whether the local administration of such natural compounds in rats can attenuate the excitability of nociceptive primary neurons in response to in vivo mechanical stimulation warrants further investigation. Prior in vitro investigations have indicated that the phytochemical quercetin exerts inhibitory effects on Nav and Cav channels and promotes the activation of Kv channels in cardiac muscle tissue [24,33]. Subsequently, our recent in vivo studies revealed that local administration of quercetin into the peripheral receptive field resulted in the suppression of excitability in rat nociceptive primary sensory neurons within the TG, possibly mediated by the inhibition of Nav channels and the activation of Kv channels at the nociceptive nerve terminals [57]. Consequently, under non-neuropathic and non-inflammatory conditions, the local anesthetic application of quercetin may provide a means of alleviating trigeminal nociceptive pain with a reduced incidence of adverse effects, thus contributing to the integration of CAM. Considering the aforementioned findings, a comparative analysis of the inhibitory effects of phytochemicals and lidocaine on the discharge frequency of nociceptive neurons elicited by noxious stimuli indicates the following relative local anesthetic efficacy: resveratrol (10 mM) = chlorogenic acid (10 mM) = genistein (10 mM) = (-)-epigallocatechin-3-gallate (10 mM) = quercetin (10 mM) > 1% lidocaine (37 mM). Consequently, it can be postulated that these phytochemicals may elicit a similar effect at a concentration roughly equivalent to one-quarter that of 1% lidocaine [98]. The rationale for this disparity lies in the fact that phytochemicals exhibit a wider array of molecular targets at nerve terminals than lidocaine, encompassing the inhibition of Nav channels, the suppression of mechanosensitive channels, and the facilitation of Kv channels (Figure 3). Nevertheless, further patch clamp investigations of TG neurons are warranted to corroborate this hypothesis [99].

Furthermore, our recent findings indicate that the local administration of (-)-epigallocatechin-3-gallate to the peripheral receptive fields diminishes the responsiveness of nociceptive primary sensory neurons located in the TG, as well as secondary SpVc neurons [58]. Upon comparing the inhibitory effects of (-)-epigallocatechin-3-gallate on primary and second-order neurons, the magnitude of inhibition observed in second-order neurons was comparatively greater in response to both non-noxious and noxious stimuli than reported in our earlier study [56]. Primary sensory neurons relay sensory information from peripheral receptive fields to second-order neurons. As a result, information convergence does not occur at the primary neuron level. Instead, second-order sensory neurons are the site of convergence, receiving input from multiple primary neurons and interneurons [82]. While the exact disparity in the inhibition rate of discharge frequency elicited by (-)-epigallocatechin-3-gallate-induced noxious stimuli is not fully elucidated, it is hypothesized that the differential convergence rate between primary and second-order neurons constitutes a major contributing factor. The observation that (-)-epigallocatechin-3-gallate exhibits a more pronounced suppressive effect on neuronal discharge frequency in second-order neurons than in first-order neurons is of notable interest, as it implies the participation of a higher center within the pain transmission pathway in augmenting the degree of pain relief.

With respect to an alternative natural compound for local anesthesia, decanoic acid (DA), a naturally occurring fatty acid, demonstrates a range of biological activities. In an investigation utilizing guinea pig duodenum and jejunum, Gwyme et al. [48] demonstrated that DA-induced motor activity is reversibly antagonized by a muscarinic antagonist, thereby implying that DA may function as an acetylcholine muscarinic 2 receptor (Ach M2R) agonist. Notably, the Ach M2R family is implicated in a multitude of significant physiological functions within both the central and peripheral nervous systems, encompassing nociception [100]. In prior research, Bernardini et al. [101] demonstrated, through electrophysiological experiments employing a rat skin–nerve preparation, that Ach M2R elicited an inhibitory or desensitizing effect on the peripheral terminals of C-nociceptors. This desensitization can be accounted for by the observation that, beyond reducing the intracellular cAMP concentration, Ach M2R also modulates low-threshold Kv channels, thereby contributing to hyperpolarization [102]. Accumulating evidence suggests the presence of Ach M2R mRNA and immunoreactivity in small- and medium-diameter TG neurons [59,103]. Corroborating these findings, our recent investigations have shown that acute application of DA elicits short-term mechanical hypoalgesia, an effect largely attributable to M2 Ach M2R-mediated membrane hyperpolarization in WDR and NS neurons of the TG and SpVc [59,60]. These observations lend support to the concept of the natural compound DA as a prospective therapeutic CAM for the management of trigeminal nociception.

### 5.2. Intravenous Anesthesia by Natural Compounds: Modulation of Nociceptive Pathways

**Figure 4:**
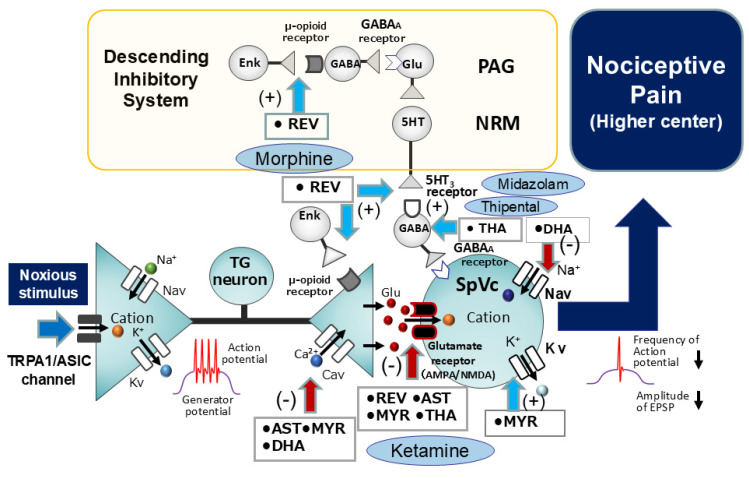
Intravenous administration of natural compounds influences nociceptive transmission. Specifically, intravenous administration of several natural co mpounds (resveratrol, theanine, docosahexaenoic acid, astaxanthin, myricetin) suppresses nociceptive neuronal transmission in the SpVc by inhibiting Cav channels and promoting the synthesis of the inhibitory neurotransmitter GABA. Resveratrol’s activation of μ-opioid receptors in the PAG increases neuronal activity via GABAergic disinhibition, which subsequently activates serotonergic neurons in the NRM. These actions inhibit synaptic transmission in the SpVc through the activation of 5-HT3 receptor-mediated GABAergic inhibition (descending inhibitory control system). Additionally, resveratrol directly activates 5-HT3 and μ-opioid receptors, further suppressing excitatory synaptic transmission in the SpVc via 5-HT3 receptor-mediated GABAergic inhibition. Intravenous administration of astaxanthin suppresses nociceptive neuronal transmission in the SpVc by inhibiting Cav channels and NMDA glutamate receptors. Similarly, intravenous administration of myricetin suppresses nociceptive neuronal transmission in the SpVc by inhibiting Cav channels and facilitating Kv channels. Finally, intravenous administration of docosahexaenoic acid suppresses nociceptive neuronal transmission in the SpVc by inhibiting both Cav and Nav channels. TG = trigeminal ganglion, PAG = periaqueductal grey, NRM = nucleus raphe magnus, SpVc = spinal trigeminal nucleus caudalis, Glu = glutamate, Enk = enkephalin, AMPA = α-amino-3-hydroxy-5-methyl-4-isoxazole propionic acid, NMDA = n-methyl-D-aspartate receptor, GABA = gamma-aminobutyric acid, 5HT = 5-hydroxytryptamine (serotonin), EPSP = excitatory postsynaptic potential, Nav = voltage-gated sodium channel, Kv = voltage-gated potassium channel, Cav = voltage-gated calcium channel, (+) = excitation, (−) = inhibition, ASIC = acid-sensing ion channel, TRPA1 = transient receptor potential ankyrin 1, Morphine = μ-opioid receptor antagonist, Ketamine = NMDA receptor antagonist, REV = Resveratrol, AST = Astaxanthin, MYR = Myricetin, DHA = Docosahexaenoic acid, THA = theanine.

Subsequent in vitro investigations conducted by Gao et al. [18] have revealed that resveratrol elicits a dose-dependent suppression of postsynaptic currents evoked by glutamate in hippocampal slice preparations. Their report elucidates that glutamate N-methyl-D-aspartate (NMDA) receptors display a higher degree of sensitivity to resveratrol than α-amino-3-hydroxy-5-methyl-4-isoxazolepropionic acid (AMPA) receptors. Additionally, resveratrol has been shown to dose-dependently inhibit long-lasting-type Cav channel currents in cardiac myocytes, an excitable cell type [17]. Drawing upon the aforementioned findings, resveratrol potentially exerts inhibitory effects on glutamate receptors located on the postsynaptic membrane and Cav channels situated on the presynaptic membrane. This action would consequently lead to the suppression of glutamatergic excitatory synaptic transmission within SpVc neurons, a crucial aspect of the glutamate-mediated synaptic transmission mechanism [61]. In a recent in vivo study [62], we examined whether the excitability of SpVc WDR neurons in response to non-noxious and noxious mechanical stimuli was modulated by the intravenous administration of resveratrol. The outcome of this investigation revealed that resveratrol dose-dependently and reversibly attenuated the firing frequency of SpVc WDR neurons elicited by both non-noxious and noxious mechanical stimuli. Moreover, resveratrol exhibited a significantly greater inhibitory effect on the firing frequency of SpVc WDR neurons in response to noxious stimuli compared to non-noxious stimuli. To elucidate the effects of resveratrol on the postsynaptic membrane, we employed intravenous administration of resveratrol and subsequently examined the neuronal activity of these SpVc WDR neurons following iontophoretic application of glutamate and NMDA using multi-barrel microelectrodes [62]. The results indicated that resveratrol inhibited the firing frequency elicited in SpVc WDR neurons by the administration of both glutamate and NMDA at a comparable rate of relative inhibition. Of particular interest was the finding that it suppressed the excitatory synaptic transmission mechanism via NMDA receptors [62] (Figure 4). To put it differently, resveratrol may possess pharmacological actions equivalent to those of ketamine, an established NMDA receptor antagonist utilized as an intravenous anesthetic with pronounced analgesic properties and a reduced incidence of respiratory depression.

It is generally established that μ-opioid receptors are expressed in neurons of the periaqueductal gray (PAG), and opioid agonists, such as morphine and enkephalin, act upon these PAG μ-opioid receptors through the activation of the descending pain inhibitory system [104,105]. As depicted in Figure 4, the descending pain inhibitory system is composed of neurons in the midbrain periaqueductal gray (PAG) and the nucleus raphe magnus, the latter containing serotonergic (5-HT) neurons [106,107]. The nociceptive jaw-opening reflex is employed as a paradigm to elucidate the inhibitory mechanisms of pain transmission through the precise modulation of electrical stimulation intensity parameters in in vitro animal studies [108,109]. Furthermore, we have demonstrated that the intravenous administration of resveratrol elicits a reversible inhibition of the nociceptive jaw-opening reflex, mediated by μ-opioid receptors, under in vivo conditions [63]. Furthermore, the inhibitory effect of intravenous resveratrol administration on the electrical stimulation-induced nociceptive jaw-opening reflex is diminished by 5-HT3 receptor inhibition (using ondansetron) and the prior administration of a GABAA receptor antagonist (bicuculline) [64]. The aforementioned experimental findings indicate that the intravenous administration of resveratrol activates the descending pain-inhibiting μ-opioid and 5-HT3 receptors under in vivo conditions, culminating in GABAergic inhibition within the SpVc region. There is strong suspicion that the activation and enhancement of the presynaptic inhibitory mechanism mediated by μ-opioid receptors in the SpVc region serves to inhibit nociceptive synaptic transmission [110]. In light of the aforementioned findings, resveratrol holds promise as an intravenous anesthetic and analgesic agent, potentially exerting effects comparable to morphine, a μ-opioid receptor agonist efficacious in the management of cancer pain, and also to barbiturate derivatives (e.g., thiopental) and benzodiazepine derivatives (e.g., midazolam), which are known for their sedative and soporific properties (Figure 4).

Concerning theanine, a natural amino acid, it has been hypothesized that it functions as a natural antagonist of glutamate receptors [37]. To investigate this hypothesis, we conducted extracellular electrophysiological studies in rats. Our findings revealed that (i) the relative extent of theanine-mediated inhibition of SpVc WDR neuronal discharge frequency was significantly more pronounced in response to noxious stimuli than to non-noxious stimuli; (ii) the mean firing frequency of SpVc WDR neurons elicited by iontophoretic application of glutamate was reduced following intravenous administration of theanine; and (iii) the mean firing frequency of SpVc WDR neurons elicited by iontophoretic application of NMDA was significantly reduced following intravenous administration of theanine [65]. Collectively, these results suggest that acute intravenous theanine administration suppresses glutamatergic excitatory postsynaptic transmission, potentially involving an NMDA receptor signaling pathway within the SpVc, through the integration of convergent inputs originating from noxious rather than non-noxious sensory information [65], analogous to the phytochemical resveratrol.

In our recent investigations, we examined the effects of intravenous administration of further natural compounds, including a carotenoid (astaxanthin), a flavonoid (myricetin), and an omega-3 fatty acid (docosahexaenoic acid). The principal findings were (i) astaxanthin, myricetin, and docosahexaenoic acid elicited a dose-dependent reduction in the firing rate of SpVc WDR neurons (i.v.) in response to both non-noxious and noxious mechanical stimuli; (ii) this inhibitory effect on discharge was reversible within a time frame of approximately 5 to 10 min; and (iii) the vehicle administered intravenously did not exert any significant influence on either the spontaneous or the evoked activity of SpVc WDR neurons [66,67,68]. Taken together, these findings indicate that, in the absence of inflammatory or neuropathic pain states, acute intravenous administration of astaxanthin, myricetin, and docosahexaenoic acid elicits a short-term inhibition of trigeminal sensory transmission, encompassing nociception, potentially through the inhibition of Cav and Nav channels and excitatory glutamate neuronal transmission, alongside the facilitation of Kv channels. The rapid anesthetic effect achieved through short-term intravenous administration, along with its sedative properties, offers the potential advantage of reducing side effects in clinical procedures where anesthesia is essential (e.g., endoscopy). However, additional studies are required to fully elucidate this hypothesis.

## 6. Modulation Mechanism of Pathological Pain by Natural Compounds

### 6.1. Natural Compounds as Local Anesthetics: Targeting Inflammatory Pain Pathways

Previous studies suggest that ASICs and TRPA1 are candidate mechanoreceptors in mammalian cutaneous nerve terminals of TG neurons. [93,94]. Furthermore, the phytochemical quercetin inhibits ASIC currents [32]. Under inflamed conditions, tissue pH falls below 6.0, and this low pH activates primary nociceptive afferents via ASICs [111]. In clinical practice, the presence of inflammation diminishes the efficacy of local anesthetics, notably, dental anesthetics [112]. Research by Fu et al. [113] has indicated that peripheral inflammation can lead to the upregulation of ASICs in TG neurons, suggesting that specific inhibitors of ASICs may exert a significant analgesic effect on orofacial inflammatory pain. These findings suggest that direct local administration of quercetin inhibits generator potentials and subsequently suppresses action potential firing in nociceptive TG neurons by inhibiting ASIC and Nav channels and opening Kv channels in inflamed tissues.

**Figure 5:**
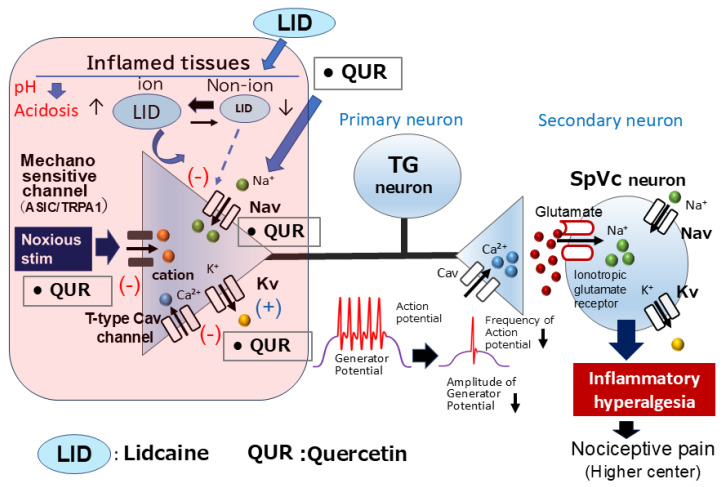
The local anesthetic effect of the natural compound quercetin, a phytochemical, is influenced by inflammatory conditions. Inflammation-induced tissue acidity lowers the pH, affecting the ionization equilibrium of lidocaine. The subsequent reduction in the non-ionized form of lidocaine diminishes its binding to Nav channels, thereby decreasing its local anesthetic efficacy in inflamed environments. In contrast, quercetin’s inhibitory action on inflamed tissues may stem from the suppression of action potential firing frequency via the inhibition of nociceptive mechanosensitive channels (ASICs), voltage-gated sodium channels (Nav), T-type calcium channels (Cav), and the activation of potassium channels (Kv). As quercetin demonstrates a significantly greater inhibitory potency on discharge frequency compared to lidocaine, it possesses a robust local anesthetic effect on inflamed tissues, suggesting its potential utility within complementary and alternative medicine. TG = trigeminal ganglion, Nav = voltage-gated sodium channel, Kv = voltage-gated potassium channel, Cav = voltage-gated calcium channel, SpVc = spinal trigeminal nucleus caudalis, ASIC = acid-sensing ion channel, TRPA1 = transient receptor potential ankyrin 1, (+) = excitation, (−) = inhibition, LID = lidocaine, QUR = Quercetin.

In a recent study, Sashide et al. [69] reported the suppressive effects of local quercetin injection into the peripheral receptive field of rats on the excitability of nociceptive primary sensory neurons within the TG. Their findings included (i) a dose-dependent (1–10 mM) and reversible inhibition of the mean firing frequency of TG neurons in response to both non-noxious and noxious mechanical stimulation by quercetin; (ii) a reversible inhibition of the mean firing frequency of inflamed TG neurons in response to mechanical stimuli by the local anesthetic 1% lidocaine (37 mM); and (iii) a significantly greater mean magnitude of inhibition of TG neuronal discharge frequency with 1 mM quercetin compared to that with 1% lidocaine [69]. Figure 5 demonstrates that the local administration of quercetin into inflamed tissue results in the suppression of excitability in nociceptive primary sensory TG neurons. This suppression is likely mediated by the inhibition of ASIC, Nav, and Cav channels, as well as the activation of Kv channels. Consequently, the local application of the phytochemical quercetin to inflamed tissues may offer a more efficacious local analgesic approach than selective Nav channel blockade, as it targets both the generation of generator potentials and action potentials in nociceptive primary nerve terminals [69]. As such, quercetin’s potential is relevant to the area of CAM. The expectation is that quercetin will emerge as a viable alternative to conventional local anesthetics, especially in inflammatory conditions, thereby expanding the scope of CAM in clinical practice. Moreover, it is anticipated that the structural analysis of quercetin analogs will be instrumental in the design of “new local anesthetics” tailored for efficacy in inflamed tissues.

### 6.2. Natural Compounds for Chronic Inflammatory Pain Relief

**Figure 6:**
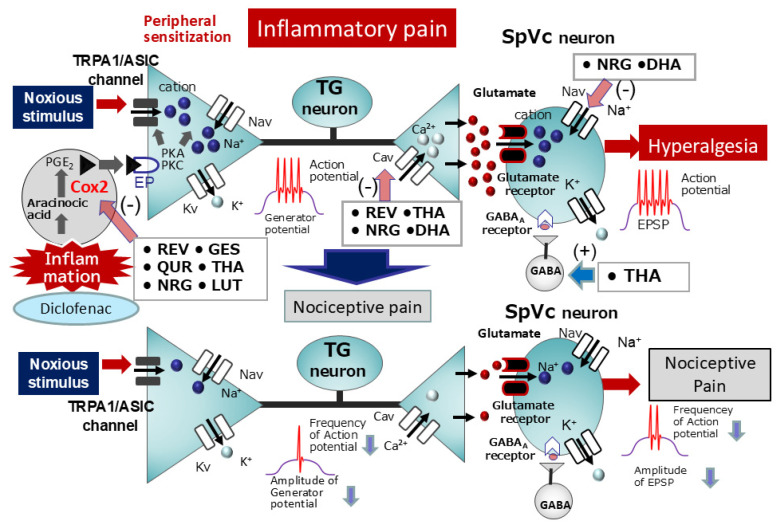
Systemic administration of natural compounds exerts inhibitory effects on inflammatory pain. In the context of peripheral inflammation, inflammatory mediators like PGE2 bind to G-protein-coupled EP receptors, initiating the activation of PKA and PKC in nociceptive peripheral terminals. This activation results in the phosphorylation of mechanosensitive channels (TRPA1, ASICs), voltage-gated sodium channels (Nav), and voltage-gated potassium channels (Kv). As a consequence, the activation threshold for transducer channels, including the TRP channel family, is lowered, and the membrane excitability of peripheral terminals is enhanced, leading to an increased frequency of action potential transmission to presynaptic central terminals within the spinal trigeminal nucleus caudalis (SpVc). This culminates in the release of substantial amounts of glutamate into the synaptic cleft, which subsequently binds to upregulated postsynaptic glutamate receptors, amplifying excitatory postsynaptic potentials (EPSPs) and triggering a high-frequency discharge of action potentials towards higher pain processing centers, establishing a state of peripheral sensitization. The systemic administration of various natural compounds, including resveratrol, isoflavone (genistein), quercetin, theanine, naringenin, lutein, and docosahexaenoic acid, mitigates mechanical inflammatory hyperalgesia associated with SpVc neuron hyperexcitability through mechanisms involving the inhibition of peripheral Cox-2 signaling cascades, modulation of voltage-gated ion channels (Cav, Nav, and Kv), and regulation of neurotransmitter synthesis. Ultimately, this action normalizes SpVc neuronal hyperactivity. TG = trigeminal ganglion, SpVc = spinal trigeminal nucleus caudalis, Nav = voltage-gated sodium channel, Kv = voltage-gated potassium channel, Cav = voltage-gated calcium channel, PGE2 = prostaglandin E_2_, EP = E-type prostanoid, ASIC = acid-sensing ion channel, TRPA1 = transient receptor potential ankyrin 1, (+) = excitation, (−) = inhibition, EPSP = excitatory postsynaptic potential, GABA = gamma-aminobutyric acid, Cox-2 = cyclooxygenase-2, Diclofenac = non-steroidal anti-inflammatory drug, REV = Resveratrol, GES = Genistein, QUR = Quercetin, THA = Theanine, NRG = Naringenin, DHA = Docosahexaenoic acid, LUT = lutein.

According to Pham-Marcou et al. [20], resveratrol regulates the Cox-2 signaling cascade, the enzyme responsible for PGE2 production, a central player in inflammatory pain development. Figure 6 shows that PGE2, an inflammatory mediator, acts on G-protein-coupled E-type prostanoid receptors of primary sensory neurons located in inflamed tissues, leading to the activation of PKA and PKC. This activation, in turn, phosphorylates and enhances the activity of nociceptive ion channels and Nav channels. The resulting increase in neuronal excitability (peripheral sensitization) is characterized by an augmented amplitude of generator potentials and a lowered excitatory threshold for Nav channels, ultimately leading to the increased transmission of action potentials to the CNS and their arrival at presynaptic terminals. The release of high concentrations of excitatory neurotransmitters via exocytosis into the synaptic cleft subsequently leads to their binding with glutamate receptors on the postsynaptic membrane. This interaction amplifies excitatory postsynaptic potentials (EPSPs) and enhances the excitability of nociceptive secondary sensory neurons, a phenomenon known as central sensitization. As a result, pain signals are efficiently transmitted to higher brain centers, contributing to hyperalgesia. Resembling NSAIDs, potent inhibitors of Cox-2, resveratrol mitigates inflammatory hyperalgesia through the inhibition of Cox-2 activity, as demonstrated by the attenuation of inflammation-induced pain-related behaviors in animals following resveratrol administration [20].

We recently investigated the potential of chronic resveratrol, genistein, and docosahexaenoic acid (DHA) administration to suppress nociceptive neuron hyperexcitability in a rat model of CFA-induced inflammatory hyperalgesia [70,71,72]. Our results showed a significant reduction in the withdrawal reflex threshold to von Frey hair mechanical stimulation in CFA-inflamed rats compared to naïve rats, which was reversed by systemic administration of resveratrol, genistein, and DHA. Two days after CFA injection, the inflammation group exhibited central sensitization, evidenced by a decreased mechanical stimulus threshold (indicative of SpVc WDR neuron hyperexcitability), increased spontaneous and evoked discharge frequencies, and enlarged receptive fields. Treatment with resveratrol, genistein, and DHA normalized all these signs of central sensitization.

In line with our previous report on resveratrol, we also found that systemic administration of quercetin effectively restored CFA-induced inflammatory hyperalgesia and SpVc WDR neuron hyperexcitability to levels indistinguishable from those of naïve rats [73]. Significantly, the inhibitory effect of quercetin (50 mg/kg, i.p.) on inflammatory hyperalgesia was comparable to that of diclofenac (50 mg/kg, i.p.), a standard NSAID [73]. While NSAIDs generally inhibit both Cox-2 at inflammatory sites and the physiologically expressed cyclooxygenase-1 (Cox-1) in the gastrointestinal tract and kidneys, increasing the risk of adverse events, our results indicate that chronic polyphenol administration can suppress SpVc WDR neuron hyperexcitability by modulating peripheral nociceptive pathways and the Cox-2 signaling cascade, thereby alleviating inflammatory hyperalgesia. Thus, polyphenols like resveratrol, isoflavones, and quercetin hold promise as alternative therapeutic agents to NSAIDs for preventing inflammatory hyperalgesia, potentially offering a better safety profile (Figure 6). This concept is also corroborated by our prior investigation [74], which demonstrated that daily administration of the phytochemical lutein for three days restored the reduced mechanical stimulation threshold in CFA-inflamed rats to control values. Lutein treatment also resolved the inflammation-induced whisker pad edema. Moreover, lutein administration normalized the increased number of Cox-2-immunoreactive cells in the whisker pads of CFA-inflamed rats. Notably, lutein significantly decreased the mean discharge frequency of SpVc WDR neurons to both non-noxious and noxious mechanical stimuli in CFA-inflamed rats. In summary, these results suggest that the phytochemical lutein attenuates inflammatory hyperalgesia associated with nociceptive SpVc WDR neuron hyperexcitability via inhibition of the peripheral Cox-2 signaling cascade [74].

To provide further evidence that the anti-inflammatory properties of natural compounds, particularly phytochemicals, are equivalent to those of NSAIDs (e.g., diclofenac), we recently employed a CFA inflammation rat model. Our behavioral and electrophysiological studies demonstrated that the phytochemical naringenin exhibits an anti-inflammatory effect comparable to diclofenac. Moreover, we found that substituting half the dose of diclofenac (DIC) with naringenin produced a similar level of anti-inflammatory activity [75]. Our study revealed the following: (i) restoration of the reduced mechanical threshold to baseline levels in inflamed rats by the second day of consistent diclofenac administration or a combination of half-dose diclofenac and half-dose naringenin; (ii) a notable reduction in the mean firing rate of SpVc WDR neurons in response to both non-noxious and noxious mechanical stimuli in inflamed rats following diclofenac or half-dose diclofenac plus half-dose naringenin administration; (iii) a notable decrease in the elevated mean spontaneous activity of SpVc WDR neurons in inflamed rats following diclofenac treatment or a combination of half-dose diclofenac and half-dose naringenin; and (iv) normalization of the enlarged mean receptive field size in inflamed rats with diclofenac or a combination of half-dose diclofenac and half-dose naringenin [75]. Our findings reveal that naringenin-mediated inhibition of SpVc neuron hyperexcitability associated with hyperalgesia was almost equivalent to that of diclofenac (50 mg/kg, i.p.). This suggests that naringenin offers comparable efficacy to diclofenac and holds promise as a therapeutic agent within CAM strategies for preventing trigeminal inflammatory mechanical hyperalgesia, potentially with fewer side effects. This result represents a significant contribution that is expected to inform the development of next-generation NSAIDs, including “specific Cox-2 inhibitors” and “new painkillers”.

### 6.3. Natural Compounds for the Relief of Ectopic Hyperalgesia Associated with Orthodontic Pain

Within the clinical domain of dentistry, pain related to orthodontic tooth movement is generally self-limiting; however, it can represent a substantial burden for certain individuals and often results in the avoidance of orthodontic therapy. In some cases, ectopic pain, including headache attributed to orthodontic tooth movement, may present with persistent pain during mastication [114]. The precise pathological mechanisms underlying these phenomena are currently unknown. While NSAID administration can provide symptomatic relief for ectopic pain, it inherently carries the risk of gastrointestinal distress and other adverse sequelae. A specific concern in orthodontic tooth movement is the potential for NSAID-induced suppression of PGE2 synthesis to lead to a reduction in osteoclastogenesis, thereby impeding the intended dental movement.

We recently investigated the efficacy of resveratrol in alleviating ectopic hyperalgesia during orthodontic tooth movement in rats with orthodontic appliances and experimentally induced tooth movement [76]. Our study revealed that resveratrol effectively reduced ectopic hyperalgesia by inhibiting the hyperexcitability of pain-transmitting SpVc WDR neurons without compromising tooth movement. These significant findings strongly suggest a promising clinical application for the pain-relieving effects of phytochemicals in the treatment of various pathological pain conditions of the trigeminal nervous system, such as headache and temporomandibular joint pain, in the future. The current understanding of ectopic hyperalgesia pathogenesis posits the involvement of chemical-mediated communication between neurons and glial cells within the TG (see review, [83]). We have previously demonstrated that the activation of sensory ganglionic satellite glial cells modulates the excitability of primary nociceptive neurons via an interleukin-1β paracrine signaling pathway following peripheral inflammation. Our findings further suggest that cytokine release may represent a critical determinant of the magnitude of trigeminal inflammatory hyperalgesia [83].

Emerging evidence suggests that trigeminal nerve injury or tissue inflammation triggers hyperexcitability in trigeminal primary afferent neurons, alongside the activation of satellite glial cells and the accumulation of macrophages [81,84,85]. These activated satellite glial cells and macrophages, in conjunction with hyperactivated TG neurons, interact via a complex interplay of mediators, receptor mechanisms, and signaling pathways [81,84,85]. This intercellular communication can result in a subsequent amplification of TG afferent neuron excitability, characterized by their enhanced excitatory input into neurons within the SpVc and the C1/C2 region of the spinal cord. Activation of the spinal trigeminal nucleus caudalis (SpVc) and the C1/C2 region triggers the release of mediators that induce neuronal hyperexcitability, leading to the activation of microglia and astrocytes. Neuron-glia communication, mediated by various molecules, is essential for the development and maintenance of neuronal hyperexcitability, a key feature of central sensitization [81,83,84,85]. Prior research indicates that polyphenol-enriched grape seed extract can modulate cellular responses to inflammatory stimuli by inhibiting glial cell activation, thereby potentially reducing the excitability of nociceptive neurons in both the TG and the SpVc [115]. While these findings raise the possibility of natural compounds modulating the neuronal hyperexcitability of SpVc neurons through diverse molecular mechanisms, the current literature provides limited evidence on the effects of natural compounds on nociceptive neuron excitability via neuron-glial interactions; further investigation is warranted to explore this potential. Future studies should prioritize the identification of specific molecular targets of natural compounds that mediate communication between neurons in sensory ganglia and between neurons and glial cells.

## 7. Functional Significance and Therapeutic Potential of Natural Compounds: Modulating Pathways for Clinical Benefit

The growing interest in CAM underscores the need for effective alternatives when Western medicine-based pharmacotherapy fails. Our recent in vivo neurophysiological studies have shown that natural compounds, encompassing phytochemicals and fatty acids like resveratrol, chlorogenic acid, isoflavones, quercetin, catechin, lutein, and theanine, target multiple molecular pathways to produce local anesthetic, intravenous anesthetic/analgesic, and anti-inflammatory pain effects. Research focused on harnessing the pain-relieving potential of natural compounds, which can circumvent the various side effects of conventional drugs, holds significant importance for the development of highly safe, non-pharmacological treatment modalities. Our in vivo research, as detailed in this review, has established that natural compounds demonstrate (i) local anesthetic properties against nociceptive pain, with an efficacy profile closely matching that of lidocaine, a standard Nav channel antagonist; (ii) intravenous anesthetic effects on nociceptive pain, similar to established analgesics such as ketamine and morphine; (iii) a potent local analgesic effect in acute inflammatory pain, exceeding that of conventional Nav channel blockers at the inflammatory site; and (iv) anti-inflammatory efficacy in chronic pain, nearly equivalent to that of the commonly employed NSAID diclofenac.

Consequently, these findings suggest that natural compounds offer a potential avenue for alleviating both nociceptive and inflammatory pain, highlighting their promising clinical utility. We expect that future research building upon these results will lead to impactful discoveries that significantly advance medical care, particularly in drug discovery for functional foods with analgesic properties, drug-free CAM therapies, and the development of safer analgesics with reduced side effects. Finally, given that the current study focused on elucidating the “discriminative aspects” of pain, future investigations should explore the influence of natural compounds on the “emotional aspects” of pain.

The International Association for the Study of Pain has recently introduced the term “nociplastic pain” to describe pain occurring in the absence of evident tissue or nerve damage, a concept encompassing what was formerly known as psychogenic pain, nonorganic pain, etc. [116]. The underlying pathophysiology of nociplastic pain is strongly associated with central nervous system dysfunction, particularly central sensitization, and the emotional component governed by the medial pain pathway is also believed to be significant [117]. Considering the influence on the emotional facets of pain and mechanisms such as central sensitization, the involvement of the medial system in the multifaceted pathology of nociplastic pain is highly probable. As demonstrated in this paper, natural compounds can reduce peripheral sensitization, a factor contributing to central sensitization in lateral pain. Therefore, future studies should explore the clinical applicability of natural compounds in alleviating nociplastic pain. To ultimately implement the research findings detailed in this paper in clinical practice, further foundational research directed towards clinical translation will be essential.

## 8. Concluding Remarks

With recent evidence highlighting the modulation of neuronal excitability, including nociceptive sensory transmission via mechanoreceptors and voltage-gated ion channels, and the inhibition of the Cox-2 cascade by natural compounds, these substances present themselves as compelling candidates for CAM. Based on the in vivo research findings from our laboratory presented in this review, we have confirmed that natural compounds possess (i) a local anesthetic effect on nociceptive pain, with efficacy almost equivalent to that of lidocaine, a commonly used Nav channel blocker; (ii) an intravenous anesthetic effect on nociceptive pain, comparable to existing analgesics such as ketamine and morphine; (iii) a local anesthetic effect on acute inflammatory pain, acting as a more potent local analgesic at the site of inflammation than existing Nav channel blockers; and (iv) an anti-inflammatory effect on chronic pain, with efficacy almost equivalent to that of the commonly used NSAID diclofenac (Figure 3, Figure 4, Figure 5 and Figure 6). Therefore, these findings suggest that natural compounds contribute to the relief of nociceptive and inflammatory pain and imply their potential for clinical application.

## Figures and Tables

**Table 1:** Modulatory effects of natural compounds on the possible molecular targets in the various tissues, including neuronal cells, under in vitro experimental conditions.

Natural Compounds	Target Molecules	Tissues	Effects	References
Resveratrol	TRPA1	Dorsal root ganglion	Inhibition	Yu et al., (2013) [14]
	Nav channel	Dorsal root ganglion	Inhibition	Kim et al., (2005) [15]
	Kv channel	Hippocampus	Facilitation	Gao et al., (2005) [16]
	Cav channel	Cardiac myocyte	Inhibition	Liew et al., (2005) [17]
	Glu receptor (AMPA/NMDA)	Hippocampus	Inhibition	Gao et al., (2006) [18]
	Cox-2	Dorsal root ganglion	Inhibition	Subbramaiah et al., (1998) [19]
Chlorogenic acid	ASICs	Dorsal root ganglion	Inhibition	Qu et al., (2014) [21]
	Kv channel	Trigeminal ganglion	Facilitation	Zhang et al., (2004) [22]
Genistein	Nav channel	Trigeminal ganglion	Inhibition	Liu et al., (2004) [23]
	Kv channel	Trigeminal ganglion	Facilitation	Liu et al., (2004) [23]
	Cav channel	Cardiac myocyte	Inhibition	Belevych et al., (2002) [24]
	Cox-2	Head cancer	Inhibition	Ye et al., (2004) [26]
		Prostate cancer	Inhibition	Swami et al., (2007) [27]
(-)-epigallocatechin-3-gallate	ASIC	Ovary cells	Inhibition	Yan et al., (2019) [28]
	Nav channel	Dorsal root ganglion	Inhibition	Kim et al., (2009) [29]
	Kv channel	Cardiac myocyte	Facilitation	Redford et al., (2012) [30]
		Central vestibular neurons	Facilitation	Jeong et al., (2005) [31]
	Cav channel	Central vestibular neurons	Inhibition	Hou et al., (2014) [34]
Quercetin	ASICs	Central vestibular neurons	Inhibition	Mukhopadhyay et al., (2017) [32]
	Nav channel	Cardiac myocyte	Inhibition	Wallace et al., (2006) [33]
	Kv channel	Coronal artery cells	Facilitation	Hou et al., (2014) [34]
	Cav channel	Coronal artery cells	Inhibition	Hou et al., (2014) [34]
	Cox-2	Cancer cells	Inhibition	Xiao et al., (2011) [35]
				Carlsen et al., (2015) [36]
Theanine	Glu receptor	Cortical neuron	Inhibition	Kakuda et al., (2002) [37]
		Neuro-muscular junction	Inhibition	Shinozaki et al., (1978) [38]
	Cox-2	Chondrocytes	Inhibition	Bai et al., (2020) [39]
Astaxanthin	Cav receptor	Cortical neurons	Inhibition	Lin et al., (2010) [40]
	Glu receptor (NMDA)	Glial cells	Inhibition	Sharma et al., (2018) [41]
Myricetin	Cav channel	Cortical neurons	Inhibition	Chang et al., (2015) [42]
	Kv channel	Hypothalamus	Facilitation	Ma et al., (2012) [43]
Docosahexaenoic acid	Cav channel	Hippocampus	Inhibition	Vreugdenhil et al., (1996) [47]
	Nav channel	Dorsal root ganglion	Inhibition	Hong et al., (2004) [44]
		Ventricular myocyte	Inhibition	Xiao et al., (1995) [45]
		Hippocampus	Inhibition	Young et al., (2000) [46]
Decanoic acid	Ach M2 receptor	Intestine	Inhibition	Gwynne et al., (2004) [48]
Naringenin	Nav channel	Dorsal root ganglion	Inhibition	Zhou et al., (2019) [49]
	Cav channel	Dorsal root ganglion	Inhibition	Zhou et al., (2019) [49]
	Glu receptor	Dorsal horn neurons	Inhibition	Zhou et al., (2019) [49]
	Cox-2	Microglia	Inhibition	Wu et al., (2016) [50]
Lutein	TRPA1	Trigeminal ganglion	Inhibition	Horvath et al., (2012) [51]
	Cox-2	Retina	Inhibition	Choi et al., (2006) [52]

ASICs = Acid sensing ionic channels, TRPA1 = transient receptor ankyrin 1, Nav = voltage-gated Na channel, Kv = voltage-gated potassium channel, Cav = voltage gated Ca channel, Cox-2 = cyclooxygenase-2, Glu = glutamate, AMPA = α-amino-3-hydroxy-5-methyl-4-isoxazole propionic acid, NMDA = n-methyl-D-aspartate, Ach = acetylcholine, M2 = muscarine receptor 2.

## Data Availability

Not applicable.

## Abbreviations

The following abbreviations are used in this manuscript:

CAM	Complementary alternative medicine
NSAIDs	Non-steroidal anti-inflammatory drugs
Cox-2	Cyclooxygenase-2
Cox-1	Cyclooxygenase-1
EP	E-type prostanoid
ASICs	Acid sensing ionic channels
TRPA1	Transient receptor potential ankyrin 1
Nav	Voltage-gated Na channel
Kv	Voltage-gated potassium channel
Cav	Voltage-gated Ca channel
Glu	Glutamate
AMPA	α-amino-3-hydroxy-5-methyl-4-isoxazole propionic acid
NMDA	n-methyl-D-aspartate, Ach = acetylcholine
Ach	acetylcholine
M_2_R	Muscarine receptor 2
PGE_2_	Prostaglandin E_2_
TMJ	Temporomandibular joint
SpVc	Spinal trigeminal nucleus caudalis
C1–C2	Upper cervical spinal cord
PAG	Periaqueductal grey
PBN	Parabrachial nucleus
VPM	Ventromedial posterior
SI	Primary somatosensory
SII	Secondary somatosensory
ACC	Anterior cingulate cortex
INS	Insula cortex
WDR	Wide dynamic range
NS	Nociceptive-specific
TG	Trigeminal ganglion
CNS	Central nervous system
TTX-S	Tetrodotoxin-sensitive
TTX-R	Tetrodotoxin-resistant
EPSP	Excitatory postsynaptic potential
DRG	Dorsal root ganglion
REV	Resveratrol
CGA	Chlorogenic acid
GES	Genistein
QUR	Quercetin
EGCG	(-)-epigallocatechin-3-gallate
DA	Decanoic acid
G	G-protein
GABA	Gamma-aminobutyric acid
5HT	5-hydroxytryptamine
AST	Astaxanthin
MYR	Myricetin
DHA	Docosahexaenoic acid
THA	Theanine
NRG	Naringenin
LUT	Lutein
DIC	Diclofenac
PKA	Protein Kinase A
PKC	Protein Kinase C

## References

[1] Rao J.K., Mihaliak K., Kroenke K., Bradley J., Tierney W.M., Weinberger M. (1999). Use of complementary therapies for arthritis among patients of rheumatologists. Ann. Intern. Med..

[2] Konvicka J.J., Meyer T.A., McDavid A.J., Roberson C.R. (2008). Complementary/alternative medicine use among chronic pain clinic patients. J. Perianesth. Nurs..

[3] Rosenberg E.I., Genao I., Chen I., Mechaber A.J., Wood J.A., Faselis C.J., Kurz J., Menon M., O’Rorke J., Panda M. (2008). Complementary and alternative medicine use by primary care patients with chronic pain. Pain. Med..

[4] Ernst E. (2003). Complementary medicine. Curr. Opin. Rheumatol..

[5] Frémont L. (2000). Biological effects of resveratrol. Life Sci..

[6] Pervaiz S. (2003). Resveratrol: From grapevines to mammalian biology. FASEB J..

[7] Shir Y., Raja S.N., Weissman C.S., Campbell J.N., Seltzer Z. (2001). Consumption of Soy Diet before Nerve Injury Preempts the Development of Neuropathic Pain in Rats. Anesthesiology.

[8] Tall J.M., Raja S.N. (2004). Dietary Constituents as Novel Therapeutics for Pain. Clin. J. Pain.

[9] Miller R.D., Katzung B.G. (1996). Local Anesthetics. Basic and Clinical Pharmacology.

[10] Brune K., Zeilhofer H., McMahon S.B., Koltzenburg M. (2006). Antipyretic Analgesics: Basic Aspects. Wall and Melzack’s Textbook of Pain.

[11] Boadas-Vaello P., Vala J.M., Verdu E. (2017). New Pharmacological Approaches Using Polyphenols on the Physiology of Neuropathic Pain. Curr. Drug Targets.

[12] Tsuchiya H. (2017). Anesthetic Agents of Plant Origin: A Review of Phytochemicals with Anesthetic Activity. Molecules.

[13] Goya S., Goyal S., Goin A.E., Alles S.R.A. (2023). Plants-Derived Natural Products Targeting Ion Channels for Pain. Neurobiol. Pain.

[14] Yu L., Wang S., Kogure Y., Yamamoto S., Noguchi K., Dai Y. (2013). Modulation of TRP Channels by Resveratrol and Other Stilbenoids. Mol. Pain.

[15] Kim H.I., Kim T.H., Song J.H. (2005). Resveratrol Inhibits Na^+^ Currents in Rat Dorsal Root Ganglion Neurons. Brain Res..

[16] Gao Z.-B., Hu G.-Y. (2005). Trans-Resveratrol, a Red Wine Ingredient, Inhibits Voltage Activated Potassium Currents in Rat Hippocampal Neurons. Brain Res..

[17] Liew R., Stagg M.A., MacLeod K.T., Collins P. (2005). The Red Wine, Resveratrol, Exerts Acute Direct Action on Gunia-Pig Ventricular Myocytes. Eur. J. Pharmacol..

[18] Gao Z.-B., Chen X.-Q., Hu G.-Y. (2006). Inhibition of Excitatory Synaptic Transmission by Trans-Resveratrol in Rat Hippocampus. Brain Res..

[19] Subbramaiah K., Chung W.J., Michaluart P., Telang N., Inoue H., Jang M., Pezzuto J.M., Dannenberg A.J. (1998). Resveratrol Inhibits Cyclooxygenase-2 Transcription and Activity in Phorbol Ester-Treated Human Mammary Epithelial Cells. J. Biol. Chem..

[20] Pham-Marcou T.A., Beloeil H., Sun X., Gentili M., Yichi D., Benoit G., Benhamou D., Mazoit J.-X. (2008). Antinociceptive Effect of Resveratrol in Carageenun-Evoked Hyperalgesia in Rats: Prolonged Effect Related to COX-2 Expression Impairment. Pain.

[21] Qu Z.-W., Liu T.-T., Qiu C.-Y., Li J.-D., Hu W.-P. (2014). Inhibition of Acid-Sensing Ion Channels by Chlorogenic Acid in Rat Dorsal Root Ganglion Neurons. Neurosci. Lett..

[22] Zhang Y.-J., Lu X.-W., Song N., Kou L., Wu M.-K., Liu F., Wang H., Shen J.-E. (2014). Chlorogenic Acid Alters the Voltage-Gated Potassium Currents of Trigeminal Ganglion Neurons. Int. J. Oral Sci..

[23] Liu L., Yang T., Simon S.A. (2004). The Protein Tyrosine Kinase Inhibitor, Genistein, Decreases Excitability of Nociceptive Neurons. Pain.

[24] Belevych A.E., Warrier S., Harvey R.D. (2002). Genistein Inhibits Cardiac L-Type Ca^2+^ Channel Activity by a Tyrosine Kinase-Independent Mechanism. Mol. Pharmacol..

[25] Huang R., Singh M., Dillon G.H. (2010). Genistein Directly Inhibits Native and Recombinant NMDA Receptors. Neuropharmacology.

[26] Ye F., Dum W.T., Yi J., Tong X., Zhang D. (2004). Inhibition of Cyclooxygenase-2 Activity in Head and Neck Cancer Cells by Genistein. Cancer Lett..

[27] Swami S., Krishnan A.V., Moreno J., Bhattacharyya R.B., Peehl D.M., Feldman D. (2007). Calciriol and Genistein Action to Inhibit the Prostaglandin Pathway: Potential Combination Therapy to Treat Prostate Cancer. J. Nutr..

[28] Yan X.G., Li W.G., Zhu J.J., Huang C., Han S.L., Jiang Q., Xu T.L., Liu J.H. (2019). Subtype-Selective Inhibition of Acid-Sensing Ion Channel 3 by a Natural Flavonoid. CNS Neurosci. Ther..

[29] Kim T.H., Lim J.-M., Kim S.S., Park M., Song J.-H. (2009). Effects of (-)Epigallocatechin-3-Gallate on Na^+^ Currents in Rat Dorsal Root Ganglion Neurons. Eur. J. Pharmacol..

[30] Redford K.E., Rognant S., Jepps T.A., Abbott G.W. (2012). KCNQ5 Potassium Channel Activation Underlies Vasodilation by Tea. Cell Physiol. Biochem..

[31] Jeong H.S., Kim Y.S., Park J.S. (2005). Modulation of Neuronal Activity by EGCG. Brain Res..

[32] Mukhopadhyay M., Singh A., Sachchidanand S., Bera A.K. (2017). Quercetin Inhibits Acid-Sensing Ion Channels through a Putative Binding Site in the Central Vestibular Region. Neuroscience.

[33] Wallace C.H.R., Baczko I., Jones L., Fercho M., Light P.E. (2006). Inhibition of Cardiac Voltages-Gated Sodium Channels by Grape Polyphenols. Br. J. Pharmacol..

[34] Hou X., Liu Y., Niu L., Cui L., Zhang M. (2014). Enhancement of Voltage-Gated K^+^ Channels and Depression of Voltage-Gated Ca^2+^ Channels Are Involved in Quercetin-Induced Vasorelaxation in Rat Coronary Artery. Planta Med..

[35] Xiao X., Shi D., Liu L., Wang J., Xie X., Kang T., Deng W. (2011). Quercetin Suppresses Cyclooxygenase-2 Expression and Angiogenesis through Inactivation of P300 Signaling. PLoS ONE.

[36] Carlsen I., Frokiaer J., Norregaard R. (2015). Quercetin Attenuates Cyclooxygenase-2 Expression in Response to Acute Ureteral Obstruction. Am. J. Physiol. Ren. Physiol..

[37] Kakuda T., Nozawa A., Sugimoto A., Nishino H. (2002). Inhibition by Theanine of Binding of [^3^H]AMPA, [^3^H]Kainate and [^3^H]MDL105,519 to Glutamate Receptors. Biosci. Biotechnol. Biochem..

[38] Shinozaki H., Ishida M. (1978). Theanine as a Glutamate Antagonist at a Crayfish Neuromuscular Junction. Brain Res..

[39] Bai H., Zhang Z., Li Y., Song X., Ma T., Liu C., Liu L., Yuan R., Wan X., Gao L. (2020). L-Theanine Reduced the Development of Knee Osteoarthritis in Rats via Its Anti-Inflammation and Anti-Matrix Degradation Actions: In Vivo and In Vitro Study. Nutrients.

[40] Lin T.Y., Lu C.W., Wang S.J. (2010). Astaxanthin Inhibits Glutamate Release in Rat Cerebral Cortex Nerve Terminals via Suppression of Voltage-Dependent Ca^2+^ Entry and Mitogen-Activated Protein Kinase Signaling Pathway. J. Agric. Food Chem..

[41] Sharma K., Sharma D., Sharma M., Sharma N., Bidver P., Prajati N., Kalia K., Tiwati V. (2018). Astaxanthin Ameliorates Behavioral and Biochemical Alterations in In-Vitro and In-Vivo Model of Neuropathic Pain. Neurosci. Lett..

[42] Chang Y., Chang C.-Y., Wang S.-J., Huang S.-K. (2015). Myricetin Inhibits the Release of Glutamate in Rat Cerebrocortical Nerve Terminals. J. Med. Food.

[43] Ma Z., Liu T. (2012). Myricetin Facilitates Potassium Currents and Inhibits Neuronal Activity of PVN Neurons. Neurochem. Res..

[44] Hong M.-P., Kim H.I., Shin Y.K., Lee C.S., Park M., Song J.-H. (2004). Effects of Free Fatty Acids on Sodium Currents in Rat Dorsal Root Ganglion Neurons. Brain Res..

[45] Xiao Y.F., Kang J.X., Morgan J.P., Leaf A. (1995). Blocking Effects of Polyunsaturated Fatty Acids on Na^+^ Channels of Neonatal Rat Ventricular Myocytes. Proc. Natl. Acad. Sci. USA.

[46] Young C., Gean P.W., Chiou L.C., Shen Y.Z. (2000). Docosahexaenoic Acid Inhibits Synaptic Transmission and Epileptiform Activity in the Rat Hippocampus. Synapse.

[47] Vreugdenhil M., Bruehl C., Voskuyl R.A., Kang J.X., Leaf A., Wadman W.J. (1996). Polyunsaturated Fatty Acids Modulate Sodium and Calcium Currents in CA1 Neurons. Proc. Natl. Acad. Sci. USA.

[48] Gwynne R.M., Thomas E.A., Goh S.M., Sjövall H., Bornstein J.C. (2004). Segmentation Induced by Intraluminal Fatty Acid in Isolated Guinea-Pig Duodenum and Jejenum. J. Physiol..

[49] Zhou Y., Cai S., Moutal A., Yu J., Comez K., Madura C.L., Shan Z., Pham N.Y.N., Sefafini M.J., Dorame A. (2019). The Natural Flavonoid Naringenin Elicits Analgesia through Inhibition of Nav1.8 Voltage-Gated Sodium Channels. ACS Chem. Neurosci..

[50] Wu L.-H., Lin C., Lin H.-Y., Liu Y.-S., Wu C.Y.-J., Tsai C.-F., Chang P.-C., Yeh W.-L., Lu D.-Y. (2016). Naringenin Suppresses Neuroinflammatory Responses through Inducing Suppression of Cytokine Signaling 3 Expression. Mol. Neurobiol..

[51] Horvath G., Szoke E., Kemeny A., Bagoly T., Deli J., Szente L., Pal S., Sandor K., Szolcsanyi J., Helyes Z. (2012). Lutein Inhibits Function of the Transient Receptor Potential A1 in Channel in Different in in Vivo and in Vitro Models. J. Mol. Neurosci..

[52] Choi J.S., Kim D., Hong Y.M., Mizuno S., Joo C.K. (2006). Inhibition of nNOS and Cox-2 Expression by Lutein in Acute Retinal Ischemia. Nutrition.

[53] Shimazu Y., Shibuya E., Takehana S., Sekiguchi K., Oshima K., Kamata H., Kairibe H., Takeda M. (2016). Local Administration of Resveratrol Inhibits Excitability of Nociceptive Wide-Dynamic Range Neurons in Rat Trigeminal Spinal Nucleus Caudalis. Brain Res. Bull..

[54] Kakita K., Tsubouchi H., Adachi M., Takehana S., Shimazu Y., Takeda M. (2017). Local Subcutaneous Injection of Chlorogenic Acid Inhibits the Nociceptive Trigeminal Spinal Nucleus Caudalis Neurons in Rats. Neurosci. Res..

[55] Yamaguchi M., Kinouchi R., Morizumi S., Shimazu Y., Takeda M. (2021). Local Administration of Genistein as a Local Anesthetic Agent Inhibits the Trigeminal Nociceptive Neuronal Activity in Rats. Brain Res. Bull..

[56] Uchino M., Sashide Y., Takeda M. (2023). Suppression of the Excitability of Rat Nociceptive Secondary Sensory Neurons Following Local Administration of the Phytochemical, (-)-Epigallocatechin-3-Gallate. Brain Res..

[57] Toyota R., Ito H., Sashide Y., Takeda M. (2023). Suppression of the Excitability of Rat Nociceptive Primary Sensory Neurons Following Local Administration of the Phytochemical Quercetin. J. Pain.

[58] Utugi S., Chida R., Yamaguchi S., Sashide Y., Takeda M. (2025). Local Administration of (−)-Epigallocatechin-3-Gallate as a Local Anesthetic Agent Inhibits the Excitability of Rat Nociceptive Primary Sensory Neurons. Cells.

[59] Noguchi Y., Matsuzawa N., Akama Y., Sekiguchi K., Takehana S., Shimazu Y., Takeda M. (2017). Dietary Constituents, Decanoic Acid Suppresses the Excitability of Nociceptive Trigeminal Neuronal Activity Associated with Hypoalgesia via Muscarinic M2 Receptor Signaling. Mol. Pain.

[60] Nakajima R., Uehara A., Takehana S., Akama Y., Shimazu Y., Takeda M. (2018). Decanoic Acid Attenuates the Excitability of Nociceptive Trigeminal Primary and Secondary Neuron Associated with Hypoalgesia. J. Pain Res..

[61] Takehana S., Sekiguchi K., Inoue M., Kubota Y., Ito Y., Yui K., Shimazu Y., Takeda M. (2016). Systemic Administration of Resveratrol Suppress the Nociceptive Neuronal Activity of Spinal Trigeminal Nucleus Caudalis in Rats. Brain Res. Bull..

[62] Takehana S., Kubota Y., Uotsu N., Yui K., Iwata K., Shimazu Y., Takeda M. (2017). The Dietary Constituent Resveratrol Suppresses Nociceptive Transmission via NMDA Receptor. Mol. Pain.

[63] Kokuba S., Takehana S., Oshima K., Shimazu Y., Takeda M. (2017). Systemic Administration of the Dietary Constituent Resveratrol Inhibits the Nociceptive Jaw-Opening Reflex in Rats via the Endogenous Opioid System. Neurosci. Res..

[64] Hirata K., Nishiki Y., Goto R., Inagaki M., Oshima K., Shimazu Y., Takeda M. (2020). Resveratrol Suppresses Nociceptive Jaw-Opening Reflex via 5HT_3_ Receptor-Mediated GABAergic Inhibition. Neurosci. Res..

[65] Takehana S., Kubota Y., Uotsu N., Yui K., Shimazu Y., Takeda M. (2017). Acute Intravenous Administration of Dietary Constituent Theanine Suppresses Noxious Neuronal Synaptic Transmission of Trigeminal Spinal Nucleus Caudalis in Rats. Brain Res. Bull..

[66] Chida R., Yamaguchi S., Utugi S., Sashide Y., Takeda M. (2024). Suppression of the Excitability of Rat Nociceptive Secondary Sensory Neurons Following Systemic Administration of Astaxanthin. Anesth. Res..

[67] Yamaguchi S., Chida R., Utugi S., Sashide Y., Takeda M. (2025). Systemic Administration of the Phytochemical, Myricetin, Attenuates the Excitability of Rat Nociceptive Secondary Trigeminal Neuron. Molecules.

[68] Takahashi H., Sashide Y., Takeda M. (2025). Systemic Administration of Docosahexaenoic Acid Suppresses Trigeminal Secondary Nociceptive Neuronal Activity in Rats. Int. J. Transl. Med..

[69] Sashide Y., Toyota R., Takeda M. (2024). Local Administration of the Phytochemical, Quercetin, Attenuates the Hyperexcitability of Rat Nociceptive Primary Sensory Neurons Following Inflammation Comparable to Lidocaine. J. Pain.

[70] Sekiguchi K., Takehana S., Shibuya E., Matsuzawa N., Hidaka H., Kanai Y., Inoue M., Kubota Y., Shimazu Y., Takeda M. (2016). Resveratrol Attenuates Inflammation-Induced Hyperexcitability of Trigeminal Spinal Nucleus Caudalis Neurons Associated with Hyperalgesia in Rats. Mol. Pain.

[71] Arakawa S., Inoue M., Kinouchi R., Morizumi S., Yamaguchi M., Shimazu Y., Takeda M. (2019). Dietary Constituents Genistein Inhibits the Hyperexcitability of Trigeminal Nociceptive Neurons Associated with Mechanical Hyperalgesia Following Orfacial Inflammation. J. Oral Biosci..

[72] Nakazaki S., Tadokoro K., Takehana S., Syoji S., Shimazu Y., Takeda M. (2018). Docosahexaenoic Acid Attenuates Inflammation-Induced Hyperexcitability of Trigeminal Spinal Nucleus Caudalis Neurons Associated with Hyperalgesia in Rats. Eur. J. Oral Sci..

[73] Itou H., Toyota R., Takeda M. (2022). Phytochemical Quercetin Alleviates Hyperexcitability of Trigeminal Nociceptive Neurons Associated with Inflammatory Hyperalgesia Comparable to NSAIDs. Mol. Pain.

[74] Syoji Y., Kobayashi R., Miyamura N., Hirohara T., Kubota Y., Uotsu N., Yui K., Shimmazu Y., Takeda M. (2018). Suppression of Hyperexcitability of Trigeminal Nociceptive Neurons Associated with Inflammatory Hyperalgesia Following Systemic Administration of Lutein via Inhibition of Cyclooxygenase-2 Cascade Signaling. J. Inflamm..

[75] Yajima S., Sakata R., Watanuki Y., Sashide Y., Takeda M. (2024). Naringenin Suppresses the Hyperexcitability of Trigeminal Nociceptive Neurons Associated with Inflammatory Hyperalgesia: Replacement of NSAIDs with Phytochemicals. Nutrients.

[76] Okubo N., Ishikawa H., Sano R., Shimazu Y., Takeda M. (2020). Effect of Resveratrol on the Hyperexcitability of Nociceptive Neurons Associated with Ectopic Hyperalgesia Induced by Experimental Tooth Movement. Eur. J. Oral Biosci..

[77] Drissi I., Woods W.A., Woods C.G. (2020). Understanding the Genetic Basis of Congenital Insensitivity to Pain. Br. Med. Bull..

[78] Cao H., Zhang Y.-Q. (2008). Spinal Glial Activation Contributes to Pathological Pain States. Neurosci. Biobehav. Rev..

[79] Scholz J., Woolf C.J. (2002). Can We Conquer Pain?. Nat. Neurosci..

[80] Cervero F., Basbaum A.I., Bushnell M.C. (2013). Pain Theories. Science of Pain.

[81] Takeda M., Matsumoto S., Sessle B.J., Shinoda M., Iwata K. (2011). Peripheral and Central Mechanisms of Trigeminal Neuropathic and Inflammatory Pain. J. Oral Biosci..

[82] Iwata K., Takeda M., Oh S.B., Shinoda M., Farah C.S., Balasubramaniam R., McCullough M.J. (2017). Neurophysiology of Orofacial Pain. Contemporary Oral Medicine.

[83] Takeda M., Takahashi M., Matsumoto S. (2009). Contribution of the Activation of Satellite Glia in Sensory Ganglia to Pathological Pain. Neurosci. Biobehav. Rev..

[84] Sessle B.J. (2021). Chronic Orofacial Pain: Models, Mechanisms, and Genetic and Related Environmental Influences. Int. J. Mol. Sci..

[85] Shinoda M., Suzuro H., Iwata K., Hayashi Y. (2022). Plastic Changes in Nociceptive Pathways Contributing to Persistent Orofacial Pain. J. Oral Biosci..

[86] Sessle B.J. (2005). Peripheral and Central Mechanisms of Orofacial Pain and Their Clinical Correlates. Minerva Anestesiol..

[87] Iwata K., Kenshalo D.R., Dubner R., Nahin R.L. (1992). Diencephalic Projections from the Superficial and Deep Laminae of the Medullary Dorsal Horn in the Rat. J. Comp. Neurol..

[88] Iwata K., Tashiro A., Tsuboi Y., Imai T., Sumino R., Morimoto T., Dubner R., Ren K. (1999). Medullary Dorsal Horn Neuronal Activity in Rats with Persistent Temporomandibular Joint and Perioral Inflammation. J. Neurophysiol..

[89] Iwata K., Miyachi S., Imanishi M., Tsuboi Y., Kitagawa J., Teramoto K., Suzuro H., Shinoda M., Kondo M., Tkada M. (2011). Ascending Multisynaptic Pathways from the Trigeminal Ganglion to the Anterior Cingulate Cortex. Exp. Neurol..

[90] Al-Khater K.M., Todd A.J. (2009). Collateral Projections of Neurons in Laminae I, III, and IV of Rat Spinal Cord to Thalamus, Periaqueductal Gray Matter, and Lateral Parabrachial Area. J. Comp. Neurol..

[91] Ito S.I. (1998). Possible Representation of Somatic Pain in the Rat Insular Visceral Sensory Cortex: A Field Potential Study. Neurosci. Lett..

[92] Harriott A.M., Gold M.S. (2009). Contribution of Primary Afferent Channels to Neuropathic Pain. Curr. Pain Headache Rep..

[93] Price M.P., McIlwrath S.L., Xie J., Cheng C., Qiao J., Tarr T.E., Sluka K.A., Brennan T.J., Lewin G.R., Welsh M.J. (2001). The DRASIC Cation Channel Contributes to the Detection of Cutaneous Touch and Acid Stimuli in Mice. Neuron.

[94] Kwan K.Y., Glazer J.M., Corey D.P., Rice F.L., Stucky C.L. (2009). TRPA1 Modulates Mechanotransduction in Cutaneous Sensory Neurons. J. Neurosci..

[95] Borzan J., Zhao C., Mayer R.A., Raja S.N. (2010). A Role for Acid-Sensing Ion Channel 3, but Not Acid-Sensing Ion Channels 2, in Sensing Dynamic Mechanical Stimuli. Anesthesiology.

[96] Kang S., Jang J.H., Price M.P., Gautam M., Benson C.J., Geong H.G., Weish M.J., Brennan T.J. (2012). Simultaneous Disruption of Mouse ASIC1a, ASIC2 and ASIC3 Genes Enhances Cutaneous Mechanosensitivity. PLoS ONE.

[97] Akopian A.N., Sivilotti L., Wood J.N. (1996). A Tetrodotoxin-Resistant Voltage-Gated Sodium Channel Expressed by Sensory Neurons. Nature.

[98] Takeda M., Shimazu Y., Rajendram R., Patel V., Preedy V.R. (2021). Dietary Constituents Act as Local Anesthetic Agents: Neurophysiological Mechanism of Nociceptive Pain (Chapter 40). The Neuroscience of Pain, Anesthetics and Analgesics: Treatments, Mechanisms, and Adverse Reactions of Anesthetics and Analgesics.

[99] Nakagawa K., Takeda M., Tsuboi Y., Kondo M., Kitagawa J., Matsumoto S., Kobayashi A., Sessle B.J., Shinoda M., Iwata K. (2010). Alternation of Primary Afferent Activity Following Inferior Alveolar Nerve Transection in Rats. Mol. Pain.

[100] Wess J., Duttaroy A., Gomeza J., Zhang W., Yamada M., Felder C.C., Bernardini N., Reeh P.W. (2003). Muscarinic Receptor Subtypes Mediating Central and Peripheral Antinociception Studied with Muscarinic Receptor Knockout Mice: A Review. Life Sci..

[101] Bernardini N., Sauer S.K., Haberberger R., Fischer M.J., Reeh P.W. (2001). Excitatory Nicotinic and Desensitizing Muscarinic (M2) Effects on C-Nociceptors in Isolated Rat Skin. J. Neurosci..

[102] Pan Z.Z., Williams J.T. (1994). Muscarine Hyperpolarizes a Subpopulation of Neurons by Activating an M2 Muscarinic Receptor in Rat Nucleus Raphe Magnus In Vitro. J. Neurosci..

[103] Dussor G.O., Helesic G., Hargreaves K.M., Flores C.M. (2004). Cholinergic Modulation of Nociceptive Responses In Vivo and Neuropeptide Release In Vitro at the Level of Primary Sensory Neuron. Pain.

[104] Gebhart G.F., Randich A., Klem W.R., Vertes R.P. (1990). Brain Stem Modulation of Nociception. Brain Stem Mechanisms of Behavior.

[105] Chieng B., Christie M.J. (1994). Hyperpolarization by Opioids Acting on μ-Receptors of a Subpopulation of Rat Periaqueductal Grey Neurons In Vitro. Br. J. Pharmacol..

[106] Tanimoto T., Takeda M., Nishikawa T., Matsumoto S. (2004). The Role of 5-HT3 Receptors in the Vagal Afferent Activation-Induced of C1 Spinal Neurons Projected from Tooth-Pulp in the Rat. J. Pharmacol. Exp. Ther..

[107] Oshima K., Takeda M., Tanimoto T., Katsuumi I., Matsumoto S. (2005). Tooth-Pulp-Evoked Rostral Spinal Trigeminal Nucleus Neuron Activity Is Suppressed by Conditioning Sciatic Nerve Stimulation in the Rats: Possible Role of 5HT3 Receptor Mediated GABAergic Inhibition. Brain Res. Bull..

[108] Mason P., Strassman A., Maciewicz R. (1985). Is the Jaw-Opening Reflex a Valid Model of Pain?. Brain Res. Rev..

[109] Takeda M., Tanimoto T., Ojima K., Matsumoto S. (1998). Suppressive Effect of Vagal Afferents on the Activity of Trigeminal Neurons Related to Jaw-Opening Reflex in Rats: Involvement of Endogenous Opioid System. Brain Res. Bull..

[110] Takeda M., Tanimoto T., Ikeda M., Kadoi J., Nasu M., Matsumoto S. (2004). Opioidergic Modulation of Excitability of Rat Trigeminal Root Ganglion Neurons Projection to the Superficial Layer of Cervical Dorsal Horn. Neuroscience.

[111] Punnia-Moorthy A. (1987). Evaluation of pH Changes in Inflammation of the Subcutaneous Air Pouch Lining in the Rat, Induced by Carrageenan, Dextran and Staphylococcus Aureus. J. Oral Pathol..

[112] Boronat López A., Peñarrocha Diago M. (2006). Failure of Locoregional Anesthesia in Dental Practice. Review of the Literature. Med. Oral Patol. Oral Cir. Bucal.

[113] Fu H., Fang P., Zhou H.-Y., Zhou J., Yu X.-W., Ni M., Zheng J.-Y., Jin Y., Chen J.-G., Wang F. (2016). Acid-Sensing Ion Channels in Trigeminal Ganglion Neurons Innervating the Orofacial Region Contribute to Orofacial Inflammatory Pain. Clin. Exp. Pharmacol. Physiol..

[114] Bergius M., Berggren U., Kiliaridis S. (2002). Experience of Pain during an Orthodontic Procedure. Eur. J. Oral Sci..

[115] Cady R.J., Hirst J.J., Durham P.L. (2010). Dietary Grape Seed Polyphenols Repress Neuron and Glia Activation in Trigeminal Ganglion and Trigeminal Nucleus Caudalis. Mol. Pain.

[116] Fitzcharles M.A., Cohen S.P., Clauw D.J., Littlejohn G., Usui C., Hauser W. (2021). Nociplastic Pain: Towards an Understanding of Prevalent Pain Conditions. Lancet.

[117] Yoo Y.-M., Kim K.-H. (2024). Current Understanding of Nociplastic Pain. Korean J. Pain.

